# Saturable human neopterin response to interferon-α assessed by a pharmacokinetic-pharmacodynamic model

**DOI:** 10.1186/1479-5876-11-240

**Published:** 2013-10-02

**Authors:** Sangil Jeon, Jae-Hyeon Juhn, Seunghoon Han, Jongtae Lee, Taegon Hong, Jeongki Paek, Dong-Seok Yim

**Affiliations:** 1 Department of Clinical Pharmacology and Therapeutics, Seoul St. Mary’s Hospital, 222, Banpodaero, Seocho-gu, Seoul, Korea; 2 LG Life Sciences Co., Ltd, LG Gwanghwamun Bldg., Sinmunno 2-ga, Jongno-gu, Seoul, Korea

**Keywords:** PK-PD modeling, IFN-α, NONMEM, Neopterin

## Abstract

**Background:**

In this study, we developed a pharmacokinetic (PK)- pharmacodynamic (PD) model of a new sustained release formulation of interferon-α-2a (SR-IFN-α) using the blood concentration of IFN-α and neopterin in order to quantify the magnitude and saturation of neopterin production over time in healthy volunteers. The SR-IFN-α in this study is a solid microparticular formulation manufactured by spray drying of a feeding solution containing IFN-α, a biocompatible polymer (polyethylene glycol) and sodium hyaluronate.

**Methods:**

The full PK and PD (neopterin concentration) datasets from 24 healthy subjects obtained after single doses of 9, 18, 27 and 36 MIU of subcutaneous SR-IFN-α were used to build the mixed-effect model using NONMEM (version 7.2) with the GFORTRAN compiler.

**Results:**

A one-compartment model with first-order elimination and a mixture of zero- and first-order absorption was chosen to describe the PK of SR-IFN-α. The time-concentration profile of neopterin, the PD marker, was described by a turnover model combined with a single transit compartment. The saturable pattern of the neopterin response blurring the dose–response relationship of SR-IFN-α was addressed by introducing the concept of the EC_50_ increasing over time.

**Conclusions:**

The PK-PD model of SR-IFN-α developed in this study has presented a quantitative tool to assess the time-course of a saturable neopterin response in humans.

## Background

Interferons are produced by peripheral blood leukocytes, fibroblasts, and activated T and NK cells in response to viral infection or other inducers including double-stranded RNA, lipopolysaccharide, micro-organisms, or endotoxins [1-3]. Based on immunological and physicochemical differences, human interferons are divided into α-, β-, and γ-interferon families, with numerous subtypes within each interferon family [4]. Because of its antiviral, antiproliferative and immunomodulating properties, recombinant interferon-α (IFN-α) has been used as a treatment for various diseases [5]. However, in the case of chronic hepatitis C, monotherapy with IFN-α has been persistently effective in only a small percentage of patients [6]. This low response rate is thought to be due to HCV genotype variation and/or the quite short half-life of IFN-α [7,8]. Combination therapy with other antiviral agents, such as ribavirin, is therefore recommended [9]. Frequent administration (3 times weekly) of IFN-α has been considered to be an additional cause for therapeutic failure of interferon due to the fact that frequent administration accelerates the formation of neutralizing antibodies and causes other adverse effects due to large variations in peak-to-trough plasma drug concentrations [10,11]. Hence, a long-acting formulation has been developed for IFN-α which can extend its effects to weeks and months.

Unlike small molecule drugs, the poor stability of protein drugs has been a hurdle to the development of long acting formulations [12]. Technologies for producing long-acting formulations of protein drugs can be roughly classified into chemical modification such as pegylation [13] or formulation changes allowing delayed release from depot sites. The new sustained release formulation of IFN-α-2a (SR-IFN-α, LG Life Sciences) used in our report is a solid microparticular formulation manufactured by the spray drying of a feeding solution containing IFN-α, a biocompatible polymer (polyethylene glycol) and sodium hyaluronate [14].

This report is based upon a first-in-human, single ascending dose trial of SR-IFN-α in healthy volunteers where the IFN-α concentration profile showed an extended release pattern through the doses studied. Blood neopterin concentrations were also measured as a biomarker demonstrating the activity of IFN-α in this first-in-human study. Neopterin is a soluble immune activation marker released from monocytes and macrophages by IFN-α [15]. Looking into the relationship between the exposure to IFN-α and neopterin in the healthy subjects’ data, we found that the magnitude of neopterin concentration changes was not clearly correlated with the dose of IFN-α. Although similar phenomena have been observed in animal experiments [16,17], this has never been reported in humans despite frequent clinical trials using neopterin as a biomarker. Because clear understanding of the relationship between exposure and response is one of the fundamental goals of early-phase exploratory clinical trials, we investigated the concentration-response relationship of IFN-α and neopterin in humans, which has never been elucidated. As results, we present a pharmacokinetic-pharmacodynamic (PK-PD) model that quantifies the peculiar time-course of neopterin responses to IFN-α in humans.

## Methods

### Inclusion and exclusion criteria

Healthy volunteers aged 18 to 45 years with BMI ranging from 19 to 29 kg/m^2^, with no clinically relevant conditions identified based on medical history, physical examination, laboratory tests or electrocardiography (ECG), were eligible for inclusion. Subjects with any history that indicated a possible alteration in IFN-α metabolism or with hypersensitivity to IFN-α were excluded. The final study enrollment was 32 subjects (Table 1).

**Table 1:** **Subject demographics**^***a***^

**Group**	**Control**	**1**	**2**	**3**	**4**
Dose	3 MIU	9 MIU	18 MIU	27 MIU	36 MIU
Number of Subjects	8	6	6	6	6
Sex	Male	Male	Male	Male	Male
Age (years)	24.0 (18 ~ 43)	21.5 (19 ~ 37)	22 (19 ~ 34)	22 (18 ~ 42)	24 (20 ~ 43)
Height (cm)	181.65 (165.5 ~ 189.0)	179.05 (171.0 ~ 191.5)	183.5 (172.0 ~ 187.0)	178.15 (168.0 ~ 191.5)	181.75 (178.0 ~ 193.0)
Weight (kg)	73.85 (69.0 ~ 82.2)	78.65 (60.3 ~ 92.4)	76.15 (63.0 ~ 86.5)	74.40 (53.8 ~ 92.6)	74.40 (70.7 ~ 98.8)

^*a*^Continuous variables are shown as median (range).

### Study design

A randomized, double-blind, active controlled, dose escalation phase I clinical study was conducted on 32 healthy subjects in the clinical pharmacology unit of the Kendle International BV, located in Utrecht, Netherlands. Subjects were randomly allocated into four groups (eight subjects per group). Within each group, six were given SR-IFN-α (test formulation) and the other two were given 3 MIU Roferon-A® (Roche, active comparator) via subcutaneous injection. The doses of SR-IFN-α allocated to groups 1, 2, 3 and 4 were 9 MIU, 18 MIU, 27 MIU, and 36 MIU, respectively.

The study was performed in compliance with the European Community rules of Good Clinical Practice (GCP), the International Conference on Harmonization (ICH) Tripartite Guidelines: Guideline for GCP, the current revision of the 'Declaration of Helsinki’ (Edinburgh, amendment October 2000). The Stichting Therapeutische Evaluatie Geneesmiddelen (STEG), an independent ethics committee, approved the protocol before execution of the trial, and all participants gave written informed consent.

### Blood sampling

For the population PK analysis, peripheral blood samples (5 mL each) were taken just prior to the injection, and 0.75, 1.5, 3, 6, 8, 10, 12, 18, 24, 30, 36, 48, 60, 72, 96, 120, 144, 168, and 192 hours after the injection. For the PD marker, the sampling times differed slightly: just prior to the injection and 3, 8, 12, 18, 24, 36, 48, 72, 96, 120, 144, 168, 192, and 264 hours after the injection. The samples were collected in light-protective tubes and stored at < -70°C.

### Assay of plasma concentrations of IFN-α and neopterin

IFN-α concentrations in the serum samples were analyzed using a commercial Human IFN-α Multi-Subtype ELISA Kit (product # 41105) with a detection limit of 12.5 pg/mL manufactured by Pestka Biomedical Laboratories, Inc. (Piscataway, NJ, USA). Neopterin concentrations in the serum samples were analyzed using a commercially-available Neopterin ELISA method (REF 40-371-25012, GenWay Biotech, Inc., San Diego, CA, USA) with a detection limit of 0.7 nmol/L and the specificity of about 99.95%.

### Population PK-PD model

Because the aim of this study was to develop a PK-PD model for SR-IFN-α, data from the active control group participants, who were given the immediate release IFN-α formulation (8 subjects), were not included in the analysis (individual plots for PK-PD models are shown in Additional file 1).

**Figure 1:**
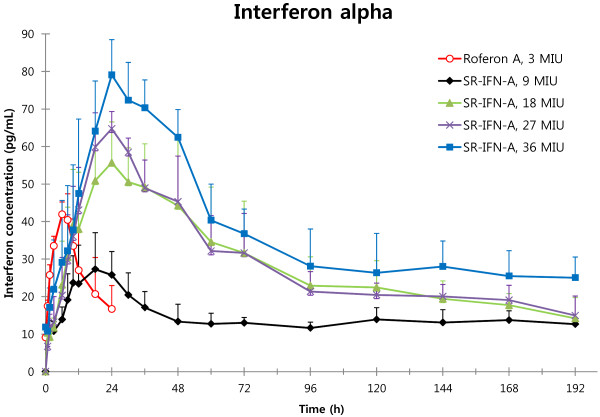
Plasma concentration–time curves of interferon-α (Mean and S.D.).

Mean plasma concentrations of IFN-α are shown in Figure 1, and non-compartmental analysis results of the PK of SR-IFN-α are summarized in Table 2. The population PK-PD analysis was performed using NONMEM (version 7.2, Icon Development Solutions, Ellicott City, MD, USA) with the GFORTRAN compiler.

**Table 2:** Non-compartmental analysis of interferon-α: Mean ± S.D. (CV%)

**Group**	**Dose**	**Formulation**	**C**_**max **_**(pg/mL)**	**Median T**_**max **_**(h)**	**AUC**_**last **_**(ng·h/mL)**
Control^*a*^ (N=8)	3 MIU	Roferon-A®	43.27 ± 9.804 (22.66%)	6	0.9664 ± 0.9322 (96.46%)
1 (N=6)	9 MIU	SR-IFN-α	28.33 ± 9.656 (34.08%)	18	2.072 ± 1.134 (54.73%)
2 (N=6)	18 MIU	SR-IFN-α	62.12 ± 15.93 (25.64%)	24	5.373 ± 1.382 (25.72%)
3 (N=6)	27 MIU	SR-IFN-α	65.73 ± 6.702 (10.20%)	24	5.544 ± 0.5509 (9.94%)
4 (N=6)	36 MIU	SR-IFN-α	80.31 ± 9.859 (12.28%)	24	7.151 ± 1.132 (15.83%)

^*a*^There was not a separate control group, but data from a total of eight subjects (two subjects in each of the four groups) who received 3 MIU of Roferon-A® were shown.

To find the model that best described the absorption profile, which showed double peaks in many subjects, first- and zero-order absorption models and their combined form, with or without lag time, were tested. Based on first-order elimination, one- and two-compartment distribution models were tested for the three absorption (first-order, zero-order and combined) processes. The Michaelis-Menten absorption and elimination models were also tested considering the potential saturable absorption or elimination using the ADVAN subroutines (Table 3).

**Table 3:** PK model development process

**Step**	**Model tested**^***a***^	**Objective function value**
1	One-compartment model with first-order absorption	2138.416
	One-compartment model with first-order absorption (with lag-time)	2130.609
2	Two-compartment model with first-order absorption	2037.527
	Two-compartment model with first-order absorption (with lag-time)	1993.071
	Two-compartment model with saturable absorption (Michaelis Menten absorption)	2140.583
3	Two-compartment model with zero-order absorption	1885.795
4	One-compartment model with a mixture of zero and first-order absorption	1879.067
	One-compartment model with Michaelis Menten elimination and a mixture of zero- and first-order absorption	2163.942

^*a*^The elimination process was assumed to follow first-order kinetics.

The coupling of the PK model to the PD model was done in a sequential manner. The population PD modeling was performed using the individual PK parameters estimated from the final PK model, which were added to the PD dataset.

Turnover models, with or without transit compartments, were compared to find the most appropriate model that explained the delayed effect of IFN-α on neopterin concentrations. The turnover model was initially selected over the effect compartment model based upon the well-known action of IFN-α stimulating the release of neopterin, and the transit compartments were tested because their usefulness was reported in a previous preclinical study [16].

**Figure 2:**
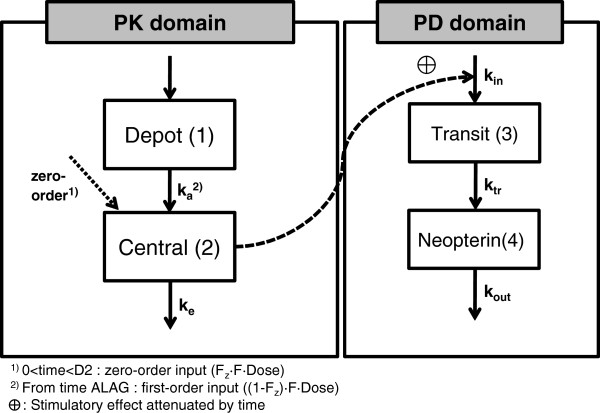
**PK-PD model structure.** The mixed absorption with one-compartment first-order elimination model (PK) and the turnover model with a single transit compartment (PD) are shown.

The differential equations for the drug effect model (model structures are shown in Figure 2) were:

$$\frac{\mathrm{dA}\left(1\right)}{\mathrm{dt}}=-K_{a}\cdot A\left(1\right)$$

$$\frac{\mathrm{dA}\left(2\right)}{\mathrm{dt}}=K_{a}\cdot A\left(1\right)-K_{e}\cdot A\left(2\right)$$

$$\frac{\mathrm{dA}\left(3\right)}{\mathrm{dt}}=K_{\mathrm{in}}\cdot E\left(C\right)-K_{\mathrm{tr}}\cdot A\left(3\right)$$

$$\frac{\mathrm{dA}\left(4\right)}{\mathrm{dt}}=K_{\mathrm{tr}}\cdot A\left(3\right)-K_{\text{out}}\cdot A\left(4\right)$$

where K_a_ and K_e_ are the absorption and elimination rate constants for IFN-α, respectively. K_in_ is the production rate of neopterin, a zero-order constant, K_tr_ is the first-order transition rate leaving the transit compartment, K_out_ is the first-order rate constant for the elimination of neopterin, and E(C) is the effect as a function of the individual predicted drug concentration, C. A(4), which is the amount existing in the 4th compartment, indicates the concentration of neopterin.

The stimulatory function for the drug effect, E(C), was a sigmoid function:

$$E\left(C\right)=1+\frac{E_{\text{max}}\cdot C^{r}}{EC_{50}{}^{r}+C^{r}}$$

where E_max_ is the maximum effect and EC_50_ is the IFN-α concentration that produces 50% of the maximum effect.

**Figure 3:**
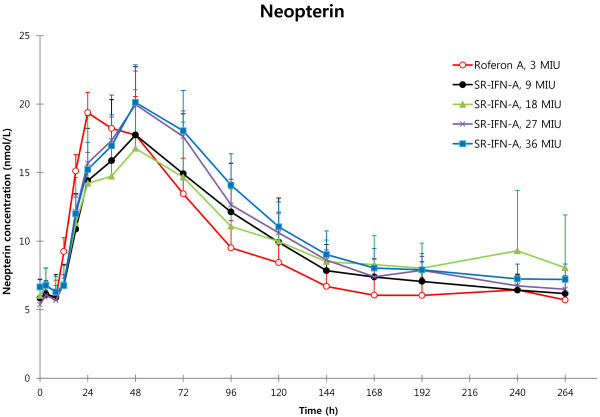
**Plasma neopterin concentration changes in response to interferon-α (Mean and S.D.).** Plasma neopterin concentrations after injection of Roferon-A® or SR-IFN-α show little difference between dose groups.

In our study, differences in the mean plasma neopterin concentrations between the groups receiving different doses of SR-IFN-α were not clearly discernible (Figure 3). To account for this phenomenon in our PD model, we incorporated the concept of time-dependent attenuation of the effect parameters [18], especially the increasing EC_50_ over time, as shown in the following equation:

$$EC_{50}=\mathrm{ECB}\times \left(1+\mathrm{CA}\times \left(1-e^{-\mathrm{CB}\times \text{TIME}}\right)\right)$$

where ECB is the baseline of EC_50_ and CA and CB are coefficients to describe the EC_50_ increase to a certain level (ECB × CA) over time. This curve is the cumulative distribution function (CDF) of an exponential distribution to EC_50_. It has advantages in explaining the concave-shaped curves in relation to time.

The first-order conditional estimation (FOCE) method with interaction was used throughout the model building. Models were selected based upon a decrease in the objective function value (OFV) of more than 3.84 (*P*-value 0.05 in an approximate *χ*2 distribution) and improvement in the individual plots, as well as other scatterplots.

A log normal distribution was assumed for inter-individual variability (η), and PK or PD parameters of the j_th_ subject (P_j_) were described as:

$$P_{j}=\mathrm{TVP}\times \text{exp}\left(\eta_{j}\right)$$

where TVP represents the typical population value of PK-PD parameters, such as clearance (CL), volume of distribution (V), absorption rate constant (K_a_), lag time (ALAG), the first-order elimination rate of serum neopterin (K_out_), and maximum stimulation effect (E_max_). The inter-individual variability eta (η) for each PK-PD parameter was assumed to follow a Gaussian distribution with a mean 0 and a variance ω^2^. Possible correlations between the inter-individual variability were also evaluated.

As for the residual error, the additive, proportional and combined forms were tested. An example of the combined error form is shown as follows:

$$Y_{\mathrm{ij}}=\text{IPRE}D_{\mathrm{ij}}+\sqrt{\epsilon_{\text{add},\mathrm{ij}}{}^{2}+{\left(\epsilon_{\text{prop},\mathrm{ij}}\times \text{IPRE}D_{\mathrm{ij}}\right)}^{2}}$$

where IPRED_ij_ is the individual predicted concentration, Y_ij_ is the measured concentration of the j_th_ individual at the i_th_ sampling time, and ϵ_ij_ is residual error. Residual errors (ϵ) include intra-individual variability, assay error and model misspecification. They were also assumed to follow a Gaussian distribution with a mean 0 and variance σ^2^.

### Covariate selection

Age, height, weight and creatinine clearance were screened as potential covariates of the parameters using Generalized Additive Modeling (GAM) implemented by Xpose version 4.2.3.

In the forward selection of covariates, variables that decreased the OFV by more than 3.84 (*P* < 0.05) and improved the inter-individual variability (omega value decrease) were selected. Covariates that did not increase the OFV more than 3.84 (*P* < 0.05) in the backward elimination step were removed from the model.

### Model evaluation

The 95% confidence intervals (CIs) for mean population PK and PD parameters were determined using a re-sampling technique based on the bootstrap method. One thousand re-sampled datasets were collected and their parameters were estimated using our population models. The models were also evaluated by visual predictive checks (VPC) using 1,000 simulated datasets.

## Results

### Population PK-PD model

**Figure 4:**
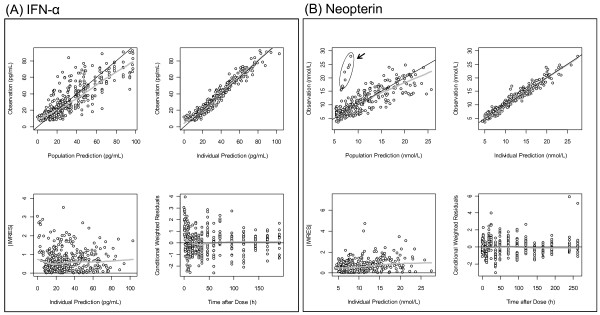
**Basic goodness of fit plots for PK and PD models. (A)** and **(B)** are goodness of fit plots for PK (IFN-α) and PD (neopterin) models, respectively. Black line, line of identity; gray line, LOESS (locally weighted regression) smooth line. The encircled dots in the PD plots represent one outlier (ID No. 2) whose IFN-α concentrations were very low, but whose neopterin concentrations were rather higher.

**Figure 5:**
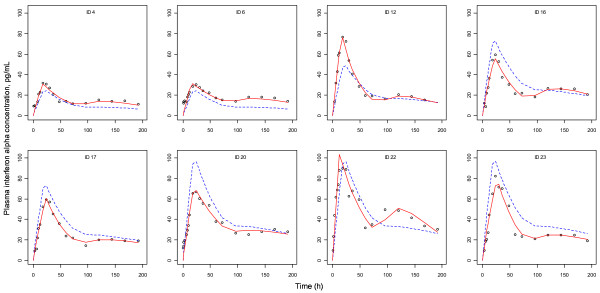
**PK curves from representative individuals showing double peaks.** Circle, observed value; dashed line, population predicted value; solid line, individual predicted value.

The results of non-compartmental PK analysis showing the trend of PK linearity of SR-IFN-α are briefly summarized in Table 2. A one-compartment model with first-order elimination and a mixture of zero- and first-order absorption best described the PK of SR-IFN-α. The delayed pattern of the time-concentration profile of neopterin was well described by the turnover model with a single transit compartment. There was no significant covariate. The structure of the final PK-PD model is shown in Figure 2. The population PK-PD parameter estimates, with corresponding standard error (SE) values, are summarized in Table 4. Basic goodness-of-fit plots are presented in Figure 4. Predicted concentration-time profiles of IFN-α and observed data from representative individuals are shown in Figure 5.

**Table 4:** Final estimates of population PK-PD parameters

**Parameter**	**Description**	**Estimate**	**%RSE**	**Bootstrap median (95% CI)**^***a***^
**Pharmacokinetics**				
CL/F (L/h)	Apparent clearance	12.2	7.39	12.5 (4.9~14.9)
V/F (L)	Apparent volume of distribution	691	6.54	712 (324~980)
D2 (h)	Duration of zero-order absorption	20.2	7.08	19.9 (16.6~23.0)
KA (h^-1^)	Absorption rate constant of first-order absorption	0.00653	16.23	0.00721 (0.0010~0.1715)
ALAG (h)	Lag time to the initiation of first-order absorption	85.7	3.92	88.1 (80.9~108.0)
RF^b^	Alternate variable for relative fraction absorbed in zero-order absorption process	0.185	55.68	0.24 (-1.17~0.94)
ω_CL_ (%)	Between subject variability of CL	26.1	35.1	24.5 (14.4~32.7)
ω_V_ (%)	Between subject variability of V	23.8	58.0	23.7 (0.4~37.6)
ω_D2_ (%)	Between subject variability of D2	35.7	27.1	34.8 (23.0~49.4)
ω_RF_ (%)	Between subject variability of RF	34.7	64.1	32.1 (0.3~54.2)
ωk_a_ (%)	Between subject variability of KA	77.0	37.1	59.5 (0.7~95.4)
σ_add_ (pg/mL)	Additive error	3.92	12.65	3.83 (-3.36~4.80)
σ_prop_ (%)	Proportional error	7.8	26.06	7.0 (-8.8~11.9)
**Pharmacodynamics**				
BASE (nmol/L)	Baseline of neopterin	5.85	4.17	5.88 (5.39~6.34)
KOUT (h^-1^)	First-order elimination rate of serum neopterin	0.0311	17.20	0.03 (0.02~0.04)
EMAX	Maximum stimulatory effect	16.1	53.19	17.45 (7.06~62.42)
GA	Hill coefficient (γ)	1.24	11.85	1.27 (1.01~2.52)
CA^c^	Coefficient	405	115.80	616.5 (27~13115)
CB^c^	Coefficient	0.0068	27.73	0.0064 (0.0014~0.0119)
ECB^c^	Baseline of EC_50_	2.17	152.07	1.66 (0.05~74.67)
MTT (h)	Mean transit time	14.6	11.37	14.5 (9.82~19.2)
ω_BASE_ (%)	Between subject variability of BASE	13.85	51.84	12.63 (3.91~20.79)
ω_CB_ (%)	Between subject variability of CB	57.31	43.31	46.21 (0.32~72.61)
ω_GA_ (%)	Between subject variability of GA	13.51	55.80	13 (0.32~36.49)
ω_ECB_ (%)	Between subject variability of ECB	21.31	172.97	21.39 (0.45~55.86)
ω_MTT_ (%)	Between subject variability of MTT	13.36	94.92	10.75 (0.55~24.21)
σ_add_ (nmol/L)	Additive error	1.14	11.05	1.11 (0.95~1.37)

^*a*^95% CIs obtained from estimation of 1000 bootstrap-resampled datasets; ^*b*^Fraction absorbed in zero-order absorption, F_z_ = e^RF^/(1+ e^RF^); Fraction absorbed in first-order absorption: 1-F_z_; ^*c*^EC50 =ECB*(1+CA*(1-e^-CB*Time^)); *RSE* Relative standard error.

### Model evaluation

**Figure 6:**
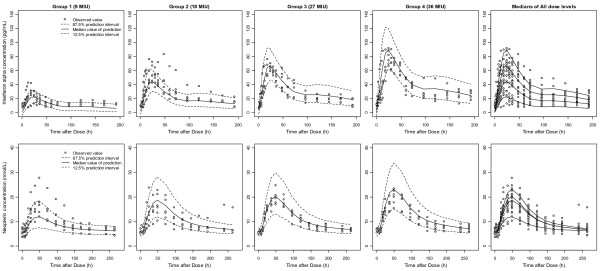
**Visual predictive checks of PK (Top) and PD (Bottom) models.** One thousand datasets (24,000 virtual subjects) were simulated using the final PK and PD parameter estimates. The simulated median (solid lines) and 75% prediction intervals (broken lines) were overlaid with observed data. The two rightmost panels presenting medians of all dose groups show dose-linearity in PK, but not in PD.

The 95% confidence intervals (CIs) for population PK-PD parameter estimates, determined using the bootstrap re-sampling method, are shown in Table 4. VPC plots simulated concentrations of 1,000 virtual datasets (nsub=1000 in the $SIMULATION block, 24,000 virtual patients) from the final model. The results from the VPC showed that the PK-PD model gave acceptable predictive performance. Curves for the 12.5th, 50th and 87.5th percentiles of concentrations were overlaid on the observed concentrations (Figure 6).

## Discussion

In this study, we presented results of PK-PD modeling of the time-concentration profiles of IFN-α and neopterin after administration of SR-IFN-α in healthy subjects.

For PK characteristics, the combined absorption model was successful in describing the double peak phenomenon. A zero-order absorption model appropriately described the initial increase in concentration, as measured by relatively frequent sampling, before reaching the maximum concentration. Subsequent second peaks (observed around 100 h after injection) were, however, best described by a first-order absorption model. One possible explanation for this double-peak phenomenon is in the method of SR-IFN-α administration: SR-IFN-α should be mixed with medium chain triglycerides (MCT) right before subcutaneous injection. Micro-droplets with various sizes might be formed in this mixing step, and their absorption rates may differ by droplet diameters. Despite differences in formulation, the CL (12.2 L/h) and V (691 L) in our report were not much different from those reported in a previous study for IFN-α (7~8 L/h and 700~850 L, respectively) in healthy subjects [19].

As for the PD model, the addition of a transit compartment before the neopterin compartment gave better outcome when compared with a simple turnover model. The transit compartment for neopterin was used to model data from monkeys [16], and we found that it is also useful in a human PK-PD model in this study. As there are a few mechanistic models that tried to explain little PD differences between dose groups (saturation of responses) [16,20,21], we tested them for our neopterin data in the preliminary PD model development step; however, none of them were successfully converged by NONMEM. Neither the precursor turnover model [20] that explained tolerance with depletion of precursor molecules, nor the turnover feedback model [21] that explained tolerance with negative feedback via a moderator compartment provided acceptable parameter estimates, and the basic goodness of fit plots were even worse than those for the descriptive model of increasing EC_50_. The inhibitory feedback model in monkeys [16] also showed similar problems. Thus, we had to use the concept of time-dependent attenuation of EC_50_ that did not include mechanistic reasoning to describe our neopterin response.

There are conflicting reports on saturation of neopterin by IFN-α in patients. A report on patients with hairy cell leukemia showed that neopterin responses and clinical efficacies after low doses (0.5-0.8 MIU/day) were similar to those after a conventional dose (3 MIU/day) in a 6-month clinical trial [22]. In another clinical trial for a controlled release formulation of IFN-α, increases in the mean AUC of neopterin were marginal among the doses tested (4.09 mM·h after 20 μg IFN-α and 6.61 mM·h after 320 μg IFN-α) [23]. The forms of IFN-α used in those studies [22,23] were non-pegylated forms, like the SR-IFN-α used in this report; however, in phase I clinical trials of pegylated IFN-α, neopterin responses were well correlated with the doses used [24,25]. Such a discrepancy in neopterin responses between non-pegylated and pegylated IFN-α suggests that the polyethylene glycol tail attached to IFN-α changed its PD parameters related to neopterin production. To the best of our knowledge, although pegylated formulations have long been used, it has never been reported that their PD profile may be different from that of non-pegylated forms.

Because neopterin is a frequently-used marker of cell-mediated immunity, it can be used to monitor the degree of immune activation in various clinical conditions, including infections, autoimmune diseases, malignancies, and other conditions [26]. Neopterin is also known to mediate the cytotoxic action of activated macrophages and dendritic cells via interactions with reactive oxygen species [27]. Thus, saturation of the neopterin response suggests that the magnitude of cytotoxic action mediated by neopterin may be similar regardless of the doses of IFN-α.

## Conclusions

We developed a human PK-PD model revealing the saturable neopterin response to IFN-α for the first time. Our model suggests that the magnitude of cytotoxic action mediated by neopterin may be similar regardless of the doses of non-pegylated IFN-α.

## Competing interests

JHJ is an employee of LG Life Sciences Co., Ltd.

## Authors’ contributions

SJ performed the PK-PD analysis and drafted the manuscript. JHJ participated in the planning and design of the clinical study and drafting the manuscript. SH, JL, TH, and JP participated in PK-PD analysis. DSY conceived the study, supervised the process of PK-PD analysis and edited the manuscript. All authors read and approved the final manuscript.

## Supplementary Material

Additional file 1 Individual plasma concentration curves of SR-IFN-α and neopterin. Click here for file

## References

[B1] Pfeffer LM Dinarello CA Herberman RB Williams BR Borden EC Bordens R Walter MR Nagabhushan TL Trotta PP Pestka S Biological properties of recombinant α-interferons: 40th anniversary of the discovery of interferons Cancer Res 1998 58 2489 2499 9635566

[B2] Stark GR Kerr IM Williams BR Silverman RH Schreiber RD How cells respond to interferons Annu Rev Biochem 1998 67 227 264 10.1146/annurev.biochem.67.1.227 9759489

[B3] Bao Q Zhao Y Niess H Conrad C Schwarz B Jauch K-W Huss R Nelson PJ Bruns CJ Mesenchymal stem cell-based tumor-targeted gene therapy in gastrointestinal cancer Stem Cells Dev 2012 21 2355 2363 10.1089/scd.2012.0060 22530882 PMC3424981

[B4] Samarajiwa SA Wilson W Hertzog PJ Type I interferons: genetics and structure 2006 Characterization and Application: The Interferons

[B5] Jonasch E Haluska FG Interferon in oncological practice: review of interferon biology, clinical applications, and toxicities Oncologist 2001 6 34 55 10.1634/theoncologist.6-1-34 11161227

[B6] Hoofnagle JH Lau D Chronic viral hepatitis — benefits of current therapies N Engl J Med 1996 334 1470 1471 10.1056/NEJM199605303342210 8618588

[B7] Lutchman G Hoofnagle JH Viral kinetics in hepatitis C Hepatology 2003 37 1257 1259 10.1053/jhep.2003.50238 12774002

[B8] Liang TJ Rehermann B Seeff LB Hoofnagle JH Pathogenesis, natural history, treatment, and prevention of hepatitis C Ann Intern Med 2000 132 296 305 10.7326/0003-4819-132-4-200002150-00008 10681285

[B9] McHutchison JG Gordon SC Schiff ER Shiffman ML Lee WM Rustgi VK Goodman ZD Ling M-H Cort S Albrecht JK Interferon alfa-2b alone or in combination with ribavirin as initial treatment for chronic hepatitis C N Engl J Med 1998 339 1485 1492 10.1056/NEJM199811193392101 9819446

[B10] Milella M Antoneill G Santantonio T Currenti M Monno L Mariano N Angarano G Dianzanl F Pastors G Neutralizing antibodies to recombinant alpha‒interferon and response to therapy in chronic hepatitis C virus infection Liver 1993 13 146 150 8336526 10.1111/j.1600-0676.1993.tb00622.x

[B11] Caliceti P Pharmacokinetics of pegylated interferons: what is misleading? Dig Liver Dis 2004 36 S334 S339 15645663 10.1016/s1590-8658(04)80002-1

[B12] Roberts M Bentley M Harris J Chemistry for peptide and protein PEGylation Adv Drug Deliv Rev 2002 54 459 476 10.1016/S0169-409X(02)00022-4 12052709

[B13] Grace M Youngster S Gitlin G Sydor W Xie L Westreich L Jacobs S Brassard D Bausch J Bordens R Structural and biologic characterization of pegylated recombinant IFN-α2b J Interferon Cytokine Res 2001 21 1103 1115 10.1089/107999001317205240 11798469

[B14] Choi SY Jeh HS Sustained release composition of protein drug 2006 *WO Patent 2,006,088,336*

[B15] Murr C Widner B Wirleitner B Fuchs D Neopterin as a marker for immune system activation Curr Drug Metab 2002 3 175 187 10.2174/1389200024605082 12003349

[B16] Hu X Olivier K Polack E Crossman M Zokowski K Gronke RS Parker S Li Z Nestorov I Baker DP In vivo pharmacology and toxicology evaluation of polyethylene glycol-conjugated interferon β-1a J Pharmacol Exp Ther 2011 338 984 996 10.1124/jpet.111.180661 21690216

[B17] Pepinsky RB LePage DJ Gill A Chakraborty A Vaidyanathan S Green M Baker DP Whalley E Hochman PS Martin P Improved pharmacokinetic properties of a polyethylene glycol-modified form of interferon-β-1a with preserved in vitro bioactivity J Pharmacol Exp Ther 2001 297 1059 1066 11356929

[B18] Colburn WA Eldon MA Simultaneous pharmacokinetic/pharmacodynamic modeling 1994 New York: Wiley

[B19] García-García I González-Delgado CA Valenzuela-Silva CM Díaz-Machado A Cruz-Díaz M Nodarse-Cuní H Pérez-Pérez O Bermúdez-Badell CH Ferrero-Bibilonia J Páez-Meireles R Pharmacokinetic and pharmacodynamic comparison of two "pegylated" interferon alpha-2 formulations in healthy male volunteers: a randomized, crossover, double-blind study BMC Pharmacol 2010 10 15 21092287 10.1186/1471-2210-10-15 PMC3001701

[B20] Licko V Ekblad E Dynamics of a metabolic system: what single-action agents reveal about acid secretion Am J Physiol Gastrointest Liver Physiol 1992 262 G581 G592 10.1152/ajpgi.1992.262.3.G581 1550245

[B21] Zuideveld KP Maas HJ Treijtel N Hulshof J van der Graaf PH Peletier LA Danhof M A set-point model with oscillatory behavior predicts the time course of 8-OH-DPAT-induced hypothermia Am J Physiol-Regul, Integ Comp Physiol 2001 281 R2059 R2071 10.1152/ajpregu.2001.281.6.R2059 11705793

[B22] Gastl G Aulitzky W Tilg H Nachbaur K Troppmair J Flener R Huber C A biological approach to optimize interferon treatment in hairy cell leukemia Immunobiology 1986 172 262 268 10.1016/S0171-2985(86)80107-3 3804368

[B23] De Leede LG Humphries JE Bechet AC Van Hoogdalem EJ Verrijk R Spencer DG Novel controlled-release lemna-derived IFN-α 2b (locteron): pharmacokinetics, pharmacodynamics, and tolerability in a phase I clinical trial J Interferon Cytokine Res 2008 28 113 122 10.1089/jir.2007.0073 18279106

[B24] Motzer RJ Rakhit A Ginsberg M Rittweger K Vuky J Yu R Fettner S Hooftman L Phase I trial of 40-kd branched pegylated interferon alfa-2a for patients with advanced renal cell carcinoma J Clin Oncol 2001 19 1312 1319 11230473 10.1200/JCO.2001.19.5.1312

[B25] Glue P Fang JW Rouzier-Panis R Raffanel C Sabo R Gupta SK Salfi M Jacobs S Pegylated interferon-α2b: pharmacokinetics, pharmacodynamics, safety, and preliminary efficacy data Clin Pharmacol Ther 2000 68 556 567 10.1067/mcp.2000.110973 11103758

[B26] Berdowska A Zwirska‒Korczala K Neopterin measurement in clinical diagnosis J Clin Pharm Ther 2001 26 319 329 10.1046/j.1365-2710.2001.00358.x 11679022

[B27] Hoffmann G Wirleitner B Fuchs D Potential role of immune system activation-associated production of neopterin derivatives in humans Inflamm Res 2003 52 313 321 10.1007/s00011-003-1181-9 14504669

